# An IL-13 Promoter Polymorphism Associated with Liver Fibrosis in Patients with *Schistosoma japonicum*

**DOI:** 10.1371/journal.pone.0135360

**Published:** 2015-08-10

**Authors:** Xin Long, Qian Chen, Jianping Zhao, Nicholas Rafaels, Priyanka Mathias, Huifang Liang, Joseph Potee, Monica Campbell, Bixiang Zhang, Li Gao, Steve N. Georas, Donata Vercelli, Terri H. Beaty, Ingo Ruczinski, Rasika Mathias, Kathleen C. Barnes, Xiaoping Chen

**Affiliations:** 1 Hepatic Surgery Center, Tongji Hospital, Tongji Medical College, Huazhong University of Science and Technology, Wuhan, Hubei province, China; 2 Johns Hopkins Asthma & Allergy Center, The Johns Hopkins University School of Medicine, Baltimore, Maryland, United States of America; 3 Division of Gastroenterology and Hepatology, Tongji Hospital, Tongji Medical College, Huazhong University of Science and Technology, Wuhan, Hubei province, China; 4 Bloomberg School of Public Health, The Johns Hopkins University, Baltimore, Maryland, United States of America; 5 Arizona Respiratory Center, University of Arizona, Tucson, Arizona, United States of America; 6 University of Rochester Medical Center, Rochester, New York, United States of America; University of Navarra School of Medicine and Center for Applied Medical Research (CIMA), SPAIN

## Abstract

The aim of this study was to determine whether two polymorphisms in the gene encoding *IL13* previously associated with *Schistosoma hematobium* (*S*. *hematobium*) and *S*. *mansoni* infection are associated with *S*. *japonicum* infection. Single nucleotide polymorphisms (SNPs) rs1800925 (IL13/-1112C>T) and rs20541 (IL13R130Q) were genotyped in 947 unrelated individuals (307 chronically infected, 339 late-stage with liver fibrosis, 301 uninfected controls) from a schistosomiasis-endemic area of Hubei province in China. Regression models were used to evaluate allelic and haplotypic associations with chronic and late-stage schistosomiasis adjusted for non-genetic covariates. Expression of IL-13 was measured in *S*. *japonicun*-infected liver fibrosis tissue and normal liver tissue from uninfected controls by immunohistochemistry (IHC). The role of rs1800925 in IL-13 transcription was further determined by Luciferase report assay using the recombinant PGL4.17-rs180092 plasmid. We found SNP rs1800925T was associated with late-stage schistosomiasis caused by *S*. *japonicum* but not chronic schistosomiasis (OR = 1.39, 95%CI = 1.02–1.91, p = 0.03) and uninfected controls (OR = 1.49, 95%CI = 1.03–2.13, p = 0.03). Moreover, the haplotype rs1800925T-rs20541C increased the risk of disease progression to late-stage schistosomiasis (OR = 1.46, p = 0.035), whereas haplotype rs1800925C-rs20541A showed a protective role against development of late-stage schistosomiasis (F = 0.188, OR = 0.61, p = 0.002). Furthermore, *S*. *japonicum*-induced fibrotic liver tissue had higher IL13 expression than normal liver tissue. Plasmid PGL4.17-rs1800925T showed a stronger relative luciferase activity than Plasmid PGL4.17-rs1800925C in 293FT, QSG-7701 and HL-7702 cell lines. In conclusion, the functional *IL13* polymorphism, rs1800925T, previously associated with risk of schistosomiasis, also contributes to risk of late-stage schistosomiasis caused by *S*. *japonicum*.

## Introduction

Schistosomiasis, a neglected parasitic disease, is highly endemic in areas along the Yangtze River in China, where nearly half a million people are infected with *S*. *japonicum* and 65 million are at risk of infection [1–4]. Over the past 50 years, great progress has been made in China in reducing disease transmission using conventional chemotherapy (Praziquantel), eradication of the intermediate snail host, and management of domestic animals including dogs, water buffalo and yellow cattle [1, 4, 5]. While these measures have reduced the number of new cases of schistosomiasis, complications associated with chronic *S*. *japonicum* infection have increased [6]. When *Schistosoma spp*. eggs are deposited in the liver, intestine, and spleen of the human host, they can persist for years, and may lead to chronic damage and liver fibrosis. If left untreated, 5%-13% of *S*. *japonicum* infections may develop in end-stage liver diseases [7, 8], including cirrhosis, portal hypertension, splenomegaly, and ascites, and eventually lead to death. It remains unknown why certain patients develop severe liver complications and others do not, despite living in a similar endemic environment.

Acute infection by *S*. *japonicum* is characterized by a Th1 immune response, with a shift toward a predominantly Th2 response during the chronic stages [9–12]. IL-13, a classic Th2 cytokine, increases during parasitic infection and plays a protective role in the host’s anti-*S*. *japonicum* immunity [10, 11, 13]. IL-13 is associated with eosinophil activation and increased levels of specific IgE against adult and larval antigens [14, 15], and enhances intestinal tract contractility, which contributes to egg expulsion during acute infection [16–18]. In addition, IL-13 acts as a profibrogenic factor involved in a protective mechanism characterized by formation of CD4^+^ T cell-driven liver granulomas, which leads to liver fibrosis [19–22]. IL-13 mediates hepatic stellate cell activation and the subsequent secretion of collagen contributes to the development of liver fibrosis [23–25]. Furthermore, increased IL-13 mRNA levels have been detected in fibrotic liver tissues in *S*. *japonicum*–infected animal models [26]. It is unknown, however, whether IL-13 expression is altered in *S*. *japonicum*-infected human liver.

The gene encoding *IL13* is located in chromosome 5q31 and includes four exons and three introns. We previously demonstrated that *IL13* SNPs rs1800925T and rs20541T were associated with low intensity of *S*. *mansoni* infection in a Brazilian population [27]. The heterozygous IL13 genotype C/T was also shown to be related to resistance to *S*. *mansoni* infection in a Kenyan population [28]. Moreover, rs1800925C was correlated with higher levels of infection by *S*. *haematobium* [29] and the haplotype rs7719175T-rs1800925C was also related with higher infection levels of *S*. *haematobium* [30]. DNA/protein interaction analysis showed that SNP rs1800925T was associated with increased binding of nuclear proteins to the polymorphic region [31], which was further confirmed in an asthma study [32]. On the other hand, Th2 polarized CD4^+^ T cells showed higher rs1800925T activity compared to non-polarized CD4+ T cells [33], suggesting it may act as the functional polymorphism. However, schistosomiasis caused by *S*. *japonicum*, the only human blood fluke that occurs in China, has not been studied for possible roles of *IL13* SNPs, especially in patients with chronic or late-stage infection.

In this study, we explored the association between *IL13* variants rs1800925 and rs20541 and both chronic and late-stage schistosomiasis in a rural Chinese population along the Yangtze River in Hubei province. We further explored the potential function of SNP rs1800925 in hepatic cell lines and embryonic kidney cell lines and measured IL-13 expression in fibrotic liver tissues from *S*. *japonicum*-infected patients and normal liver tissues from uninfected controls.

## Materials and Methods

### The study population

We recruited 947 participants from an *S*. *japonicum*-endemic area in Jiangling and Gongan counties along the Yangtze River in Hubei province, China, between August and October 2011. Clinical stages were defined according to the criteria established by the Chinese Ministry of Health [34, 35]. ‘Chronic schistosomiasis’ was defined as a history of water contact, a positive fecal examination (Kato-Katz or miracidium hatching test) [36–38] and a positive serology test including Indirect Hemagglutination Assay (IHA), ELISA Assay and circum Oval Precipitating Test (COPT), and no symptoms or mild symptoms (chronic diarrhea and or mild abdominal pain) as well as the absence of liver fibrosis confirmed by ultrasonography. ‘Late-stage schistosomiasis’ was defined as a documented history of *S*. *japonicum* infection in the past and presentation with clinical features of portal hypertension, including splenomegaly, hypersplenism, ascites, esophageal and gastric varices [34, 35]. In this study, 339 adult subjects were diagnosed as late-stage whose hepatic fibrosis was graded according to WHO guidelines [39], and 307 subjects were defined as chronic without hepatic fibrosis in the presence of a *S*. *japonicum* infection history exceeding 10 years. An additional 301 adult subjects with no history of infection, living in the same endemic area, were selected as a healthy control group. All subjects were unrelated, and were documented as permanent residents in the endemic area for more than 17 years and reported regular contact with infected water through agricultural and daily activities.

Fifteen paraffin-embedded *S*. *japonicum*-induced liver fibrosis tissues confirmed by histopathologic diagnosis from biopsies of patients with late-stage schistosomiasis during surgery and 15 normal liver tissues from the margin of hepatic hemangioma were selected from Hepatic Surgery Center, Tongji Hospital, Tongji Medical College, Huanzhong University of Science and Technology. Fibrotic liver tissues were from different patients from the same *S*. *japonicum*-endemic area who had undergone surgery of pericardial devascularization and splenectomy to treat end-stage liver diseases caused by *S*. *japonicum*. At the end of the surgery, tissue (2cmx2cmx2cm) at the edge of the liver was resected for a biopsy. Normal liver tissues were from patients outside *S*. *japonicum*-endemic area with hepatic hemangioma who received surgery for hepatic hemangioma resection. Normal liver tissue was resected from the margin of hemangioma specimen after surgery.

All research protocols were approved by Institutional Review Boards (IRBs) of Tongji hospital, Tongji Medical College, Huazhong University of Science and Technology and IRBs of Johns Hopkins University School of Medicine as well as according to ethic guidelines of NIH (National Institute of Health). All the participants in this study provided informed written consent.

### DNA isolation and SNP selection

We used EDTA.k2- anticoagulant tubes (Becton, Dickinson and Company, NJ, USA) to preserve 5 ml peripheral blood donated from participants. DNA was extracted using the Auto FlexiGene Star (AUTOGEN, INC., Holliston, MA, USA) with Autogen’s FlexiGene Blood extraction kit according to the manufacturer’s protocol. DNA qualities ware determined by a Thermo Scientific NanoDrop 2000, with concentrations >60 ng/μl and purity = 1.7–1.9.

### Genotyping

*IL13* SNPs rs1800925 and rs25041 were selected for genotyping using custom-designed primers and probes for the TaqMan Allelic Discrimination Assay (Applied Biosystems, Foster City, CA, USA) as previously described [40, 41] [27]. Reactions were set up in total of 5 μl on 384-well plates in 2.5ul TaqMan Genotyping Master Mix (Applied Biosystems, Part No. 4371357) with 10 ng DNA, 1 μM of each primer, and 0.2 μM of each probe. The thermal cycling reactions were run as following protocol: 95°C for 10 minutes, 95°C for 15 seconds with 40 circulation, 60°C for 1 minute with 40 circulation and 4°C for 30 minutes. Then the final reactions were analyzed on a 7900HT Sequence Detection System (Applied Biosystems) with Applied Biosystems Genotype software (SDS system, version 2.4).

A custom-designed Illumina OPA for the BeadXpress Reader System and the GoldenGate Assay with VeraCode Bead technology (Illumina, San Diego, CA) was used to determine gender and relatedness and to identify potential duplicates and Mendelian inconsistencies as previously described [41–44].

### Immunohistochemistry (IHC)

Paraffin-embedded liver tissues from 15 *S*. *japonicum* infected patients with late-stage liver disease and 15 patients with hepatic hemangioma were first placed in 65°Cincubator for 30 minutes to deparaffinating, then transferred into xylene for 10 minutes, dipped into 100% absolute ethanol, 90% ethanol, 80% ethanol, 70% ethanol, and washed by distilled water and PBS for hydrating. Liver tissues immerged in citrate buffer were heated in microwave on high for 8 minutes and low for 10 minutes for heat-induced epitope retrieval. Liver tissues were washed by distilled water and PBS and then immerged into 3%H_2_O_2_ in the dark room for blocking endogenous peroxidase. 5% BSA (Bovine Serum Albumin, Sigma-Aldrich, USA) was used to block non-specific epitope. 60 μl primary rabbit anti-human IL13 polyclonal antibody (A2089, Abclonal, Cambridge, MA, USA) was added to each liver sample in dilution 1:200 and the same amount of rabbit IgG isotype (A7016, Beyotime, Shanghai, China) was set as negative control for each slide with incubation at room temperature for 1 hour. Each slide was incubated with 60 μl second antibody complex (DAKO, Denmark) for 30 minutes at room temperature after three times washing and followed by DAB (DAKO, Denmark) dyeing for 48 seconds. The slides were then stained by hematoxylin for 30 seconds, differentiated by hydrochloric acid alcohol for 5 seconds and dipped into diluted ammonia water for 8 seconds. All slides were rinsed in 70% ethanol, 80% ethanol, 90% ethanol, 100% absolute ethanol and xylene one by one. Neutral balsam was used to enclose the liver tissue before covered by cover glass for microscopic examination.

The protocol for scoring slides has been described previously [45, 46]. Briefly, the total score of each slide was calculated as (score of staining intensity) x (score of number of positive staining cells). Staining intensity was graded as follows: 0, negative; 1, light yellow; 2, light brown; 3, brown. The number of positive cells was scored as 0(0–10%), 1 (11%-25%), 2 (26%-50%), 3 (51%-75%), 4 (76%-100%). The scoring was performed independently by three pathologists without clinical knowledge of the patients’ status. We observed all the hepatic cells were dyed positive so we only use scores of staining intensity to conduct analysis.

### Western blot analysis

Frozen liver tissues were ground into powder in liquid nitrogen and then lysed using radio immuno-precipitation assay (RIPA) (P00I3D, Beyotime Institute of Biotechnology, Jiangsu, China) containing protease and phosphatase inhibitors Cocktail (Roche, Switzerland). BCA (P0011, Beyotime Institute of Biotechnology, Jiangsu, China) was used to measure concentrations of the proteins. 30 μg tissue lysate was loaded on 10% SDS-PAGE (sodium dodecyl sulfate-polyacrylamide gels) (#G2003, Wuhan Goodbio Technology CO.LTD, Wuhan, China) and then electronically transferred onto PVDF membranes (Roche, Switzerland). 5% non-fatty milk in TBS (Tris-buffer saline) was used to block the membranes for 1 hour at room temperature and then the blots were reacted with primary antibodies at 4°C overnight: Rabbit anti-IL-13, 1:200 (BA3724-2, BOSTER, Wuhan, china); Mouse anti-GAPDH, 1:3000 (Promoter, Wuhan, China). The membranes were washed three time with TBST (TBS plus 0.1% Tween-20) and reacted with horseradish peroxidase (HRP)-conjugated secondary antibodies for 1h at 37°C. The immunoreactive signals were detected by chemiluminescence system and protein bands were analyzed by Quantity One software (Bio-Rad Laboratories, CA, USA) [47, 48]

### Cell lines

Human embryonic kidney cell line 293FT was a kindly gift from Dr. Zhanguo Zhang (Hepatic Surgery Center, Tongji Hospital, Huazhong University of Science and Technology). Human hepatic cell line QSG-7701was bought from the Type Culture Collection of the Chinese Academy of Sciences (GNHu7, Shanghai, China) and Human hepatic cell line HL-7702 was obtained from China Center for Type Culture Collection (3115CNCB00277, CCTCC, Wuhan, China) [46]. All cell lines were maintained in Dulbecco's Modified Eagle Medium (DMEM, Gibco, CA, USA) with 10% Fetal Bovine Serum (FBS, Gibco, USA) under the condition of 5% CO2 and 37°C.

### Luciferase activity assay

To evaluate the function of rs1800925, we first cloned plasmid construct pGL4.17- rs1800925 C from the *IL13* SNP rs1800925CG DNA template using forward primer 5’-GGGTACCTCTGATGTAGCCATCTGTGCC-3’ with KpnI restriction enzyme locus (underlined sequence) as well as protective base G and reverse primer 5’-GGCTAGCTGGCAGCTTTTATAGGCCCAA-3’ with NheI restriction site (underlined sequence)as well as protective base G. Kpnl and NheI (FD0542 and FD0974, Thermo Scientific, Waltham, Massachusetts, USA) were used to digest the above PCR products and plasmid pGL4.17 containing firefly luciferase reporter gene (VXP0440, Promega, Madison, WI, USA) and the digested products were ligated properly. The ligated products were confirmed by sequencing. Then we used pGL4.17- rs1800925 C as the template to perform overlap PCR to make site mutation plasmid construct pGL4.17-rs1800925T by forward primer 5’-TGGAGGACTTCTAGGAAAATGAGGGAAGAGCAGGAAAAGG-3’ and reverse primer 5’-CCTTTTCCTGCTCTTCCCT CATTTTCCTAGAAGTCCT CCA -3’ (underlined bases encoding rs1800925T). The final products were also determined by sequencing.

Human hepatic cells QSG-7701 (10^5^) and HL-7702 (10^5^) as well as human embryo kidney cells 293FT (10^5^) were added in 24-well plates and transfected with 0.45 μg of reporter plasmid and plasmid pRL-TK (0.05 μg) (VQP0126, Promega) as reference carrying renilla luciferase reporter gene using 0.5μl X-tremeGene HP DNA Transfectant (06366236001, Roche, Basle, Switzerland) when grown to 50–70% confluence. Luciferase activity was detected using dual luciferase reporter assay system (E1910, Promega) according to the manufacturer’s recommendations. We conducted three independent transfection experiments for each of the cell lines and set in triplicates for each plasmid construct. Relative luciferase activity was analyzed by setting the luciferase activity of empty PGL4.17 vector as 1

### Statistical analysis

Deviations from Hardy-Weinberg equilibrium (HWE) at each locus were analyzed by default the exact test using PLINK v.1.07 (http://pngu.mgh.harvard.edu/~purcell/plink/) [49, 50]. Paired linkage disequilibrium between these two *IL13* SNPs was tested by Haploview software v.4.2 (http://www.broadinstitute.org/scientific-community/science/programs/medical-and-population-genetics/haploview/haploview) [51]. Logistic regression analysis adjusted for age, gender, HBV-infection and alcohol consumption under an additive model was performed for allelic association analysis using R v.3.2.1 (http://cran.r-project.org/bin/windows/base/) and for haplotypic association analysis using PLINK (http://pngu.mgh.harvard.edu/purcell/plink/). Comparison of scores for IHC staining intensity between *S*. *japonicum*-induced liver fibrosis tissues and normal liver tissues was performed using Mann-Whitney U test by Prism 5.0 (GraphPad Software, La Jolla, CA, USA). Relative luciferase activity was shown as Mean± SEM and t test was used to compare relative luciferase activity between plasmid pGL4.17-rs1800925T and pGL4.17-rs1800925C in three cell lines by Prism 5.0.

## Results

### General clinical characteristics of Chinese population

947 subjects from a *S*. *japonicum*-endemic area in Hubei province of China were included and characterized as chronically infected with *S*. *japonicum*, late-stage schistosomiasis (liver disease), or uninfected. General clinical characteristics are summarized in Table 1. Late-stage cases were more likely to be male (74.3%) compared to chronic cases (58.3%, p<0.001) and uninfected controls (41.5%, p<0.001). Chronic cases were similarly more likely to be male (p<0.001). Significant differences according to age were observed across the three groups. These results suggest older age and male gender were the risk factors for the host developing chronic and late-stage schistosomiasis after infected with *S*. *japonicum*. Chronic cases presented greater proportion of HBV infection (15.3%) compared to healthy controls (8.3%, p = 0.0084); however, there was no difference in HBV infection ratio between late-stage cases (13.0%) and the chronic cases (p = 0.3957) or late-stage cases and healthy controls (p = 0.059). We observed chronic cases had a greater proportion of reported alcohol consumption (46.0%) compared to late-stage cases (31.2%, p<0.001) and healthy controls (31.2%, p<0.001).

**Table 1:** General characteristics of healthy control, chronic and late-stage study groups.

Variable	Controls (n = 301)	Chronic cases (n = 307)	Late-stage cases (n = 339)	*P*1	P2	*P*3
Gender (male %)	41.5%	58.3%	74.3%	<0.001	<0.001	<0.001
Age (year)	42.3±11.3	53.2±10.8	55.9±10.2	<0.001	0.002	<0.001
IHA (%)	0	100	98.8	<0.001	0.158	<0.001
HBV infection (%)	8.3	15.3	13.0	0.008	0.396	0.059
Alcohol consumption (%)	31.2	46.0	31.2	<0.001	<0.001	0.992

P1: Chronic cases versus Control cases, P2: Late-stage cases versus Chronic cases, P3: Late-stage cases versus Control cases.

IHA: Indirect Hemagglutination Assay

HBV: Hepatitis B Virus

Age was presented as mean ±standard deviation (SD)

### General characteristics of *IL13* variants in Chinese population

**Fig 1:**
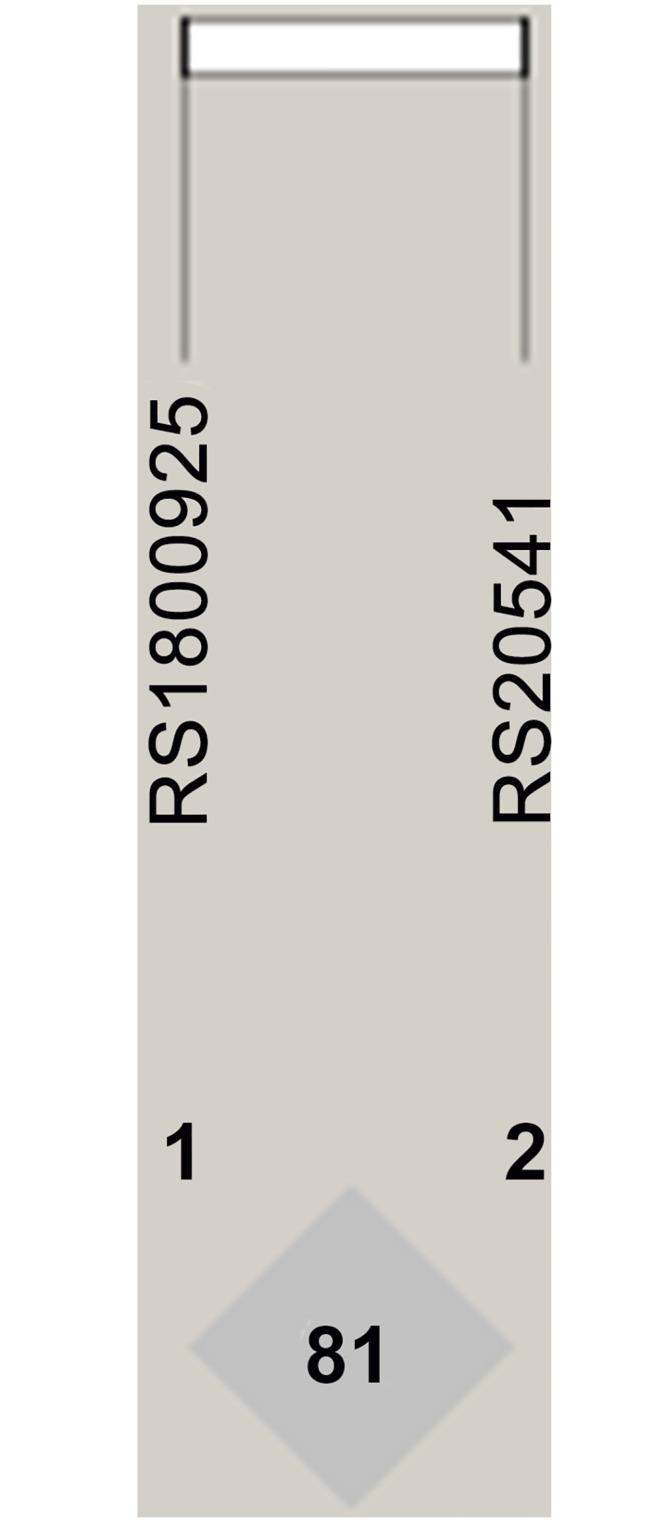
Pairwise linkage disequilibrium (LD) of 2SNPS in IL13 gene.

Tests for association between the two *IL13* markers rs1800925 and rs20541 in these Chinese samples were performed. Both markers were in Hardy-Weinberg equilibrium (S1 Table). Because both markers were relatively common in this population (minor allele frequency (MAF) for rs1800925T = 0.170; MAF rs20541A = 0.332) (Table 2), was similar to previous findings from Asian population (S2 Table), an additive model for genotypic differences was used. Linkage disequilibrium (LD) between alleles at rs1800925 and rs20541 based on Gabriel’s criteria [51], was R^2^ = 0.81 (Fig 1).

**Table 2:** General characteristics of *IL13* SNPs in Chines study samples.

SNP marker	Chromosomal position*	Gene	Functional position	Minor/major allele	MAF
rs1800925	5:131992809	IL13	Upstream-gene	T/C	0.170
rs20541	5: 131995964	IL13	Missense	A/G	0.332

* From hg19.

### Allelic association between IL13 SNPs and chronic and late-stage *Schistosoma japonicum* infection

Tests for association between IL13 SNPs and *S*. *japonicum* phenotypes is summarized in Table 3. After adjusting for age, sex, HBV-infection and alcohol consumption, we observed rs1800925T was associated with an increased risk of the development of late-stage disease when cases were compared to chronically infected patients (OR = 1.39, 95%CI = 1.02–1.91, p = 0.03) and uninfected controls (OR = 1.49, 95%CI = 1.03–2.13, p = 0.03) (Table 3). However, rs20541A was not associated with late-stage disease when compared with chronically infected patients (OR = 0.85, 95%CI = 0.65–1.11, p = 0.23) and uninfected controls (OR = 0.91, 95%CI = 0.67–1.24, p = 0.56).

**Table 3:** Allelic association of *IL13* SNPs with chronic and late-stage *S*. *japonicum infection*.

SNP marker	Minor allele	Chronic cases vs controls		Late-stage cases vs chronic cases		Late-stage cases vs controls	
		OR (95% CI)	*P*	OR (95% CI)	*P*	OR (95% CI)	*P*
rs1800925	T	1.05 (0.74–1.49)	0.80	1.39 (1.02–1.91)	0.03	1.49 (1.03–2.13)	0.03
rs20541	A	0.96 (0.72–1.72)	0.81	0.85 (0.65–1.11)	0.23	0.91 (0.67–1.24)	0.56

### Associations between the rs1800925T-rs20541A haplotype and chronic and late-stage *S*. *japonicum infection*

We performed haplotype analyses between the two IL13 SNPs and both chronic *S*. *japonicum* infection and late-stage schistosomiasis, adjusting for age, sex, HBV-infection and alcohol consumption (Table 4). Haplotype rs1800925T-rs20541A demonstrated an increased risk of progressing into late-stage disease (OR = 1.46, p = 0.035), and, haplotype rs1800925C-rs20541A suggested a protective role against late-stage disease (OR = 0.61, p = 0.002). When comparing late-stage cases with uninfected controls, the haplotype rs1800925C-rs20541A appeared to protect healthy individuals against late-stage schistosomiasis (OR = 0.65, p = 0.032). The haplotype rs1800925T-rs20541G showed a trend to increase the risk of developing into late-stage *S*. *japonicum* compared to healthy controls No association was observed when comparing chronic cases with uninfected controls.

**Table 4:** Haplotypic association of *IL13* SNPs with chronic and late-stage *S*. *japonicum* infection.

Haplotype		F1	F2	F3	Chronic cases vs controls		Late-stage cases vs chronic cases		Late-stage cases vs controls	
rs1800925	rs25041				OR(95%CI)	*P*	OR(95%CI)	*P*	OR(95%CI)	*P*
T	A	0.149	0.123	0.163	0.95(0.64–1.39)	0.777	1.46(1.03–2.08)	0.035	1.36(0.93–1.99)	0.116
C	A	0.188	0.220	0.155	1.01(0.70–1.46)	0.958	0.61(0.45–0.84)	0.002	0.65(0.44–0.96)	0.032
T	G	0.011	0.025	0.020	2.41(0.74–7.81)	0.142	0.92(0.42–2.03)	0.838	3.42(0.87–13.50)	0.079
C	G	0.652	0.632	0.662	0.96(0.72–1.28)	0.790	1.14(0.89–1.46)	0.306	1.00(1)	0.989
					Omnibus *p* = 0.157		Omnibus *p* = 0.021		Omnibus *p* = 0.251	

F1, frequency of controls; F2, frequency of chronic cases; F3, frequency of late-stage cases.

### IL-13 expression elevated in *S*. *japonicum*-induced fibrotic liver tissue

**Fig 2:**
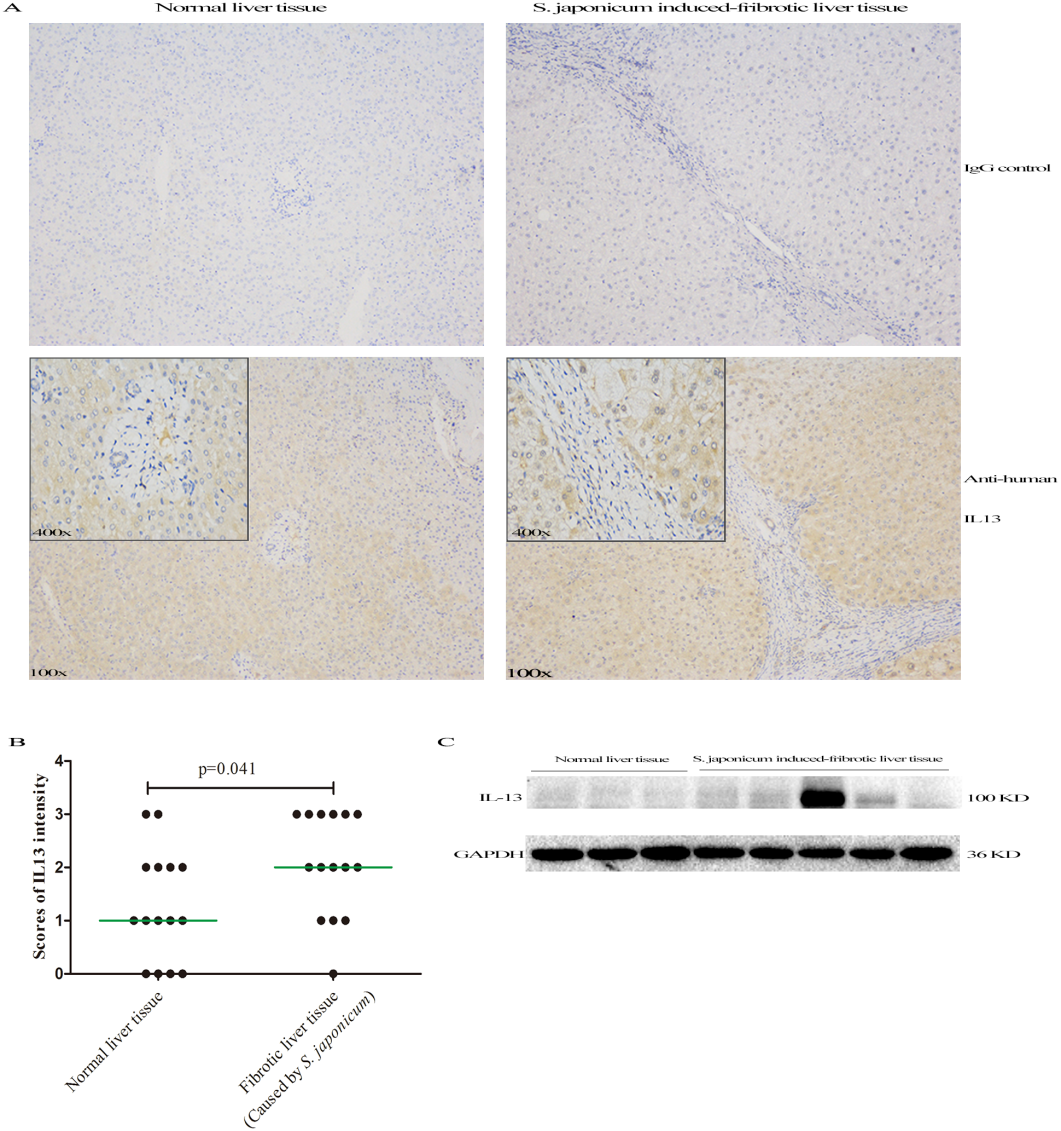
IL-13 protein was determined by IHC and western blot. (A) Representative images of normal liver tissues and *S*.*japonicum*-induced fibrotic liver tissues (Images were captured at 100x and 400x with scale bar of 100 μm). (B) Comparison of scores of ST2 staining intensity betwee *S*. *japonicum*-induced fibrotic liver tissues and normal liver tissues. (C) IL-13 protein was detected in liver tissue lysates by western blots.

Because the presence of liver fibrosis differentiates the late-stage cases from chronic cases or healthy controls and *IL13* SNP rs1800925 was associated with higher risk of liver fibrosis, we further tested whether IL-13 expression was enhanced in liver fibrosis tissue. Using IHC, we detected IL13 expression in 15 *S*. *japonicum*-induced fibrotic liver tissues and 15 normal liver tissues from the margin of hepatic hemangioma obtained from surgery. Our results showed IL-13 was expressed in all the hepatic cells and *S*. *japonicum*-induced fibrotic liver tissues had a stronger IL-13 expression than normal liver tissues (Fig 2A). IL-13 intensity was then scored for each group, indicating that IL-13 expression was increased in *S*. *japonicum*-induced fibrotic liver tissue compared to normal liver tissue (p = 0.041) (Fig 2B). Consistently, we observed the elevated IL-13 protein levels in fibrotic liver tissues by western blot compared to control liver tissues (Fig 2C).

### Functional evaluation of the *IL13* rs1800925 polymorphism

**Fig 3:**
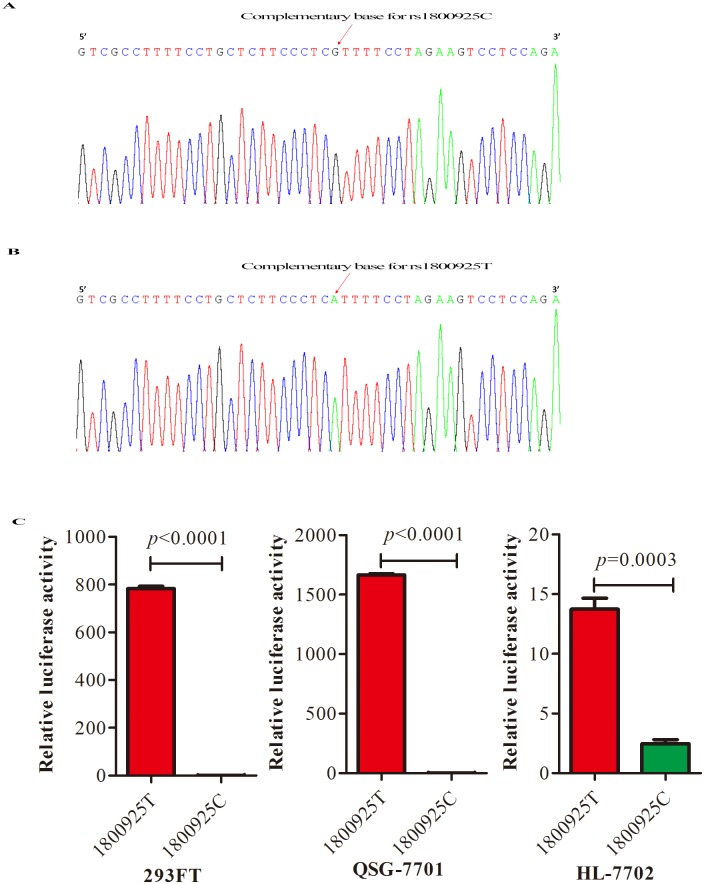
Relative luciferase activity of plasmid construct of PGL4.17-rs1800925T and PGL4.17-rs1800925C in 293FT, QSG-7701 and HL-7702 cell lines. (A) Complementary base sequence of target DNA in plasmid PGL4.17-rs1800925C. (B) Complementary base sequence of target DNA in plasmid PGL4.17-rs1800925T constructed by overlap PCR. (C) Relative luciferase activity of plasmid construct of PGL4.17-rs1800925T and PGL4.17-rs1800925C in 293FT, QSG-7701 and HL-7702 cell lines. (Rs1800925C indicates plasmid PGL4.17-rs1800925C and rs1800925T indicates plasmid PGL4.17-rs1800925T).

We next examined whether the rs1800925 variant could be a functional polymorphism for IL-13 expression in the liver. We generated luciferase report constructs pGL4.17-rs1800925C (Fig 3A) and pGL4.17-rs1800925T (Fig 3B), and the luciferase assay was used to evaluate the IL-13 transcriptional ability. Our data confirmed construct pGL4.17-rs1800925C and pGL4.17-rs1800925T presented promoter activity (Fig 3C), further verifying rs1800925 located around the promoter area of IL13 gene. We then transfected the plasmid construct into two human hepatic cell lines (QSG-7701 and HL-7701) and one human embryonic kidney cell line (293FT). All three cell lines transfected with PGL4.17-rs1800925T plasmid showed higher relative luciferase activity than the cell lines transfected with pGL4.17-rs1800925C (cell line 293FT, p<0.0001; cell line QSG-7701, p<0.001; cell line HL-7702, p = 0.0003) (Fig 3C). These results demonstrated rs1800925T increased the promoter activity of the *IL13* gene and thus may induce IL-13 protein expression in the liver.

## Discussion

In this study, we demonstrate a functional polymorphism in *IL13*, rs1800925 (IL13/-1112C>T), previously associated with risk of schistosomiasis, also increased the risk of development of late-stage schistosomiasis. Although a second functional polymorphism previously associated with schistosomiasis, rs20541 ((IL13R130Q)), was not associated with risk in this sample, the IL13 haplotype rs1800925T-rs20541A and rs1800925T-rs20541G were associated with a greater risk of progressing into late-stage schistosomiasis among patients infected with *S*. *japonicum*. In addition, rs1800925C-rs20541A appears to be protective from chronic infection and the development of late-stage schistosomiasis. Furthermore, rs1800925T showed higher transcription activity in hepatic cell lines and embryo kidney cell line than SNP rs1800925C. We also confirmed IL-13 protein levels were up-regulated in S. japonicum-induced liver fibrosis tissues compared to normal liver tissues.

Our findings showed rs1800925T was a risk allele for the severe infection of *S*. *japonicum* (late-stage schistosomiasis) rather than a protective loci for *S*. *mansoni* and *S*. *haematobium* infections as previously reported [27–31]. Furthermore, we found no association between rs1800925 and chronic infection of *S*. *japonicum* in our study. The discrepancy may be due to heterogeneity between the study populations; for example, different stages of infection among the populations. Of note, the two previous reports of associations with these functional *IL13* polymorphisms included subjects with acute infection [27, 29], whereas our study group of chronically infected subjects did not include subjects during the acute phase of infection. Importantly, the Chinese population we studied was from an endemic area where acute infection has been reduced through various modifications (vector control, prophylactic medication), while the chronic or the end-stage infection becomes more common, representing a condition in which the egg counts alone could no longer be an accurate diagnostic approach due to its low sensitivity [52]. Another difference includes the methods used to define schistosomiasis, we selected our chronic schistosomasis patients by previous history and recent results of indirect hemagglutination assay, a conventional and widely-used technique for screening *S*. *japonicum* infection in endemic area in China [53–56], which could not reflect the intensity of *S*. *japonicum* infections [52]. Moreover, our study focused only on *S*. *japonicum*, whereas the other two reports focused on *S*. *haematobium* [29] and *S*. *mansoni* [27].

Our study suggests, for the first time, a functional polymorphism in *IL13* is associated with higher protein levels in fibrotic liver tissue resulting from *S*. *japonicum* infection These data implicate IL-13 in the development and progression of liver fibrosis in the context of *S*. *japonicum* infection. Our findings are supported by several animal experiments. For example, *S*. *japonicum*-infected mice develop liver fibrosis with higher IL-13 mRNA and a large amount of collagen deposition, which could be relieved by IL-13 blockader. Furthermore, *IL13* knock-out mice infected with *S*. *japonicum* showed less eosinophil and smaller CD4^+^ T-driven granulomas and were protected from the development of liver fibrosis [23, 26, 57–60]. The underlying mechanism are thought to involve IL-13 inducing hepatic stellate cell activation and subsequent secretion of collagens leading to tissue fibrosis [61]. Consistent with previous human studies [62], here we also detected the increased, IL-13 protein levels in *S*. *japonicum*-induced fibrotic liver tissues compared to control liver tissue. Further supporting a role for IL-13 in tissue fibrosis, experiments using soluble worm antigen preparation (SWAP) or soluble egg antigen (SEA) stimulated peripheral blood mononuclear cells (PBMC) showed that IL-13 was produced and subsequently facilitated liver fibrosis [63]. Furthermore, peri-portal fibrosis resulted from *S*. *mansoni* was proved to be intensely associated with IL-13 produced by PBMC upon SEA activation [64]. Taken together with our data, these findings suggest that IL-13 plays a role in the development of hepatic fibrosis after *S*. *japonicum* infection.

Our current study also demonstrated the *IL13* SNP rs1800925T allele is associated with increased risk of liver fibrosis. This was further confirmed by our functional study of rs1800925 using luciferase activity assay *in vitro*. Our data suggest the rs1800925T variant had greater transcription activity than variant rs1800925C, which was consistent with previous SNP rs1800925 EMSA studies showing that rs1800925T could attract other transcription factors to this region and elevate IL-13 production [31, 32]. These findings suggest that controlling IL-13 expression may be a potential therapy for *S*. *japonicum-* induced liver fibrosis.

In conclusion, *IL13* functional polymorphism rs1800925T may increase the risk of liver fibrosis by *S*. *japonicum* by elevating IL-13 expression in the liver.

## Supporting Information

S1 Table Hardy-Weinberg equilibrium for IL13 SNPs in Chinese population. (PDF)

S2 Table Minor allele frequencies of *IL13* SNPs in Asia population. (PDF)
