# Supplementary material for: An IL-13 Promoter Polymorphism Associated with Liver Fibrosis in Patients with Schistosoma japonicum
Source: PLoS One. 2015 Aug 10;10(8):e0135360. doi: 10.1371/journal.pone.0135360 (PMC4530950; doi:10.1371/journal.pone.0135360)
Supplement: S1 Table — (PDF) [file pone.0135360.s001.pdf]

S1 Table. Hardy-Weinberg equilibrium for IL13 SNPs in Chinese population.

**SNP marker**	**Allele1**	**Allele2**	**HWE *P* value**
rs1800925	T	C	0.63
rs20541	A	G	0.05

HWE indicates Hardy-Weinberg equilibrium
