# Supplementary material for: An IL-13 Promoter Polymorphism Associated with Liver Fibrosis in Patients with Schistosoma japonicum
Source: PLoS One. 2015 Aug 10;10(8):e0135360. doi: 10.1371/journal.pone.0135360 (PMC4530950; doi:10.1371/journal.pone.0135360)
Supplement: S2 Table — (PDF) [file pone.0135360.s002.pdf]

S2 Table. Minor allele frequencies of *IL13* SNPs in Asia population.

SNP marker	Allele	Minor allele frequency			
		CHB	CHS	JPT	All
rs1800925	T	0.180	0.215	0.157	0.185
rs20541	A	0.325	0.385	0.303	0.339

Abbreviations, CHB, Han Chinese in Beijing; CHS, Southern Han Chinese; JPT, Japanese in Tokyo. This data is from Ensembl (http://www.ensembl.org/index.html)
